# Relationship between Structure and Rheology of Hydrogels for Various Applications

**DOI:** 10.3390/gels7040255

**Published:** 2021-12-09

**Authors:** Gorjan Stojkov, Zafarjon Niyazov, Francesco Picchioni, Ranjita K. Bose

**Affiliations:** Department of Chemical Engineering, Product Technology, University of Groningen, Nijenborgh 4, 9747 AG Groningen, The Netherlands; g.stojkov@student.rug.nl (G.S.); z.niyazov@student.rug.nl (Z.N.); f.picchioni@rug.nl (F.P.)

**Keywords:** rheology, hydrogel, polymer, application

## Abstract

Hydrogels have gained a lot of attention with their widespread use in different industrial applications. The versatility in the synthesis and the nature of the precursor reactants allow for a varying range of hydrogels with different mechanical and rheological properties. Understanding of the rheological behavior and the relationship between the chemical structure and the resulting properties is crucial, and is the focus of this review. Specifically, we include detailed discussion on the correlation between the rheological characteristics of hydrogels and their possible applications. Different rheological tests such as time, temperature and frequency sweep, among others, are described and the results of those tests are reported. The most prevalent applications of hydrogels are also discussed.

## 1. Introduction

### 1.1. Background and Motivation

**Figure 1:**
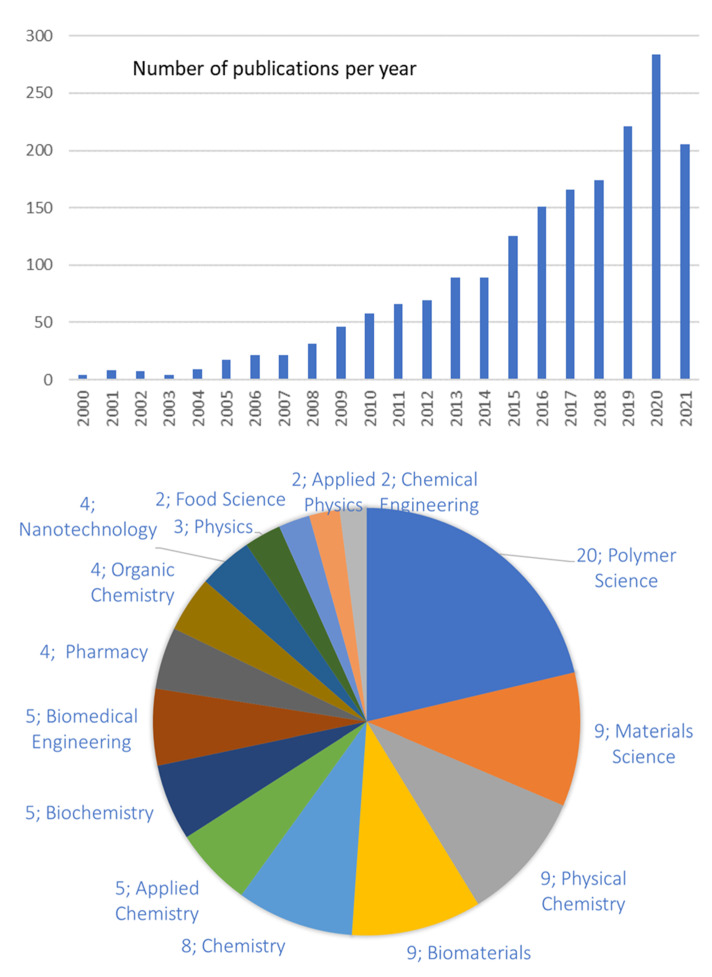
**Top**: Number of scientific publications in the last 20 years on rheology of hydrogels. **Bottom**: disciplinary areas related to the publications. Source: ISI Web of Science, November 2021, search keywords: “rheology” AND “hydrogels”.

The broad range of applications highlighted above stem from the versatility of hydrogels in terms of their chemical structures as well as properties. This is clearly reflected in the academic interest on this subject. The number of publications (Figure 1, top) in the last 20 years nicely illustrates this point. Additionally, the broad range of disciplinary areas related to these publications clearly dovetails the kaleidoscopic variety of applications. About 2% (34 out of 1865) of the publications considered are actually reviews on the topics, mostly related to very specific application areas and/or chemical compositions. A knowledge of the rheology of hydrogels helps in understanding the relationship between their chemical structure and macroscopic behavior. Furthermore, this understanding is crucial to developing hydrogels with a targeted range of properties for any specific application. To the best of our knowledge, a general helicopter view on the subject of hydrogels’ rheology has only been used for review papers published more than 10 years ago [1,2]. In this rather fragmented scientific context, this paper aims to provide the reader with a general overview of rheological tools currently used to characterize hydrogels and their structure.

### 1.2. Basic Concepts and Structure of the Paper

Gels can be placed between the materials in solid and liquid states. Among all kinds of gels, hydrogels, which are three dimensional hydrophilic structures fabricated with both natural and synthetic polymers [3,4], constitute an important class. Owing to their polymeric crosslinking, hydrogels can behave like solids and have viscoelastic properties. These characteristics render hydrogels a useful material in biomedical applications [5,6,7,8]. Different crosslinking methods can be employed to obtain these polymeric structures, the major classifications being physical crosslinking and covalent crosslinking.

In this review, we start with a description of the different crosslinking structures possible in hydrogel networks. This is followed by a detailed report and evaluation of the rheological tests used in investigating the structure-property relationships. Subsequently, we discuss the possible applications of hydrogels reported in the literature. Finally, we end with a perspective on current work and potential future of this topic.

## 2. Types of Hydrogel Networks

**Figure 2:**
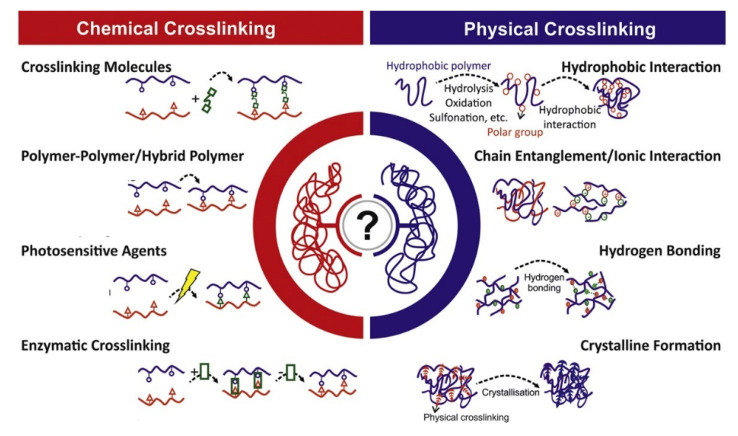
Schematic representation of various types of physical and chemical crosslinking in hydrogels (adapted from [22], CC-BY license).

In order to elucidate the relationship between structure and rheological properties, this section will provide a classification of hydrogels based on the kind of crosslinking as this is a crucial factor when analyzing the structure of a hydrogel. Generally speaking, we distinguish between physical and chemical crosslinking strategies. The first are mainly characterized by their reversible character, thus allowing the dispersion of mechanical stresses through several mechanisms at the molecular level, for example the breakage and formation of hydrogen bonds. On the other hand, chemical crosslinks yield general more rigid structures characterized by mechanical properties typical of rigid 3D networks. An overview of these classes together with general concepts of interpenetrating networks and composites hydrogels is provided in the following section. Figure 2 schematically shows the structure of each of these hydrogel types.

### 2.1. Physical Hydrogel 

Physical hydrogels are one of the weak types of hydrogels because their structure consists of the physical crosslinking of different polymer chains. The polymer chains can entangle and create transient junctions, which result in reversibility by external stimuli. The strength of the hydrogels can be attributed to different types of bonds such as hydrogen bonds [9,10,11], ionic bonds [12,13,14], hydrophobic [15,16,17,18], and hydrophilic interactions [19,20,21].

A common crosslinking method is through the formation of hydrogen bonds [10,23]. In the case of a high fraction of hydrogen bonding, a stronger hydrogel formation occurs. A good example of this is the hydrogen bonding present in polyacrylamide and poly(dodecyl glyceryl itaconate) (PGDI). In this case, polyacrylamide adsorbs to a bilayer of PDGI [3]. Hydrogels can also be formed through ionic interactions [24,25]. This is achieved through the interaction of ionizable polymers with cations. The cations can be of different valence levels and can directly affect the level of crosslinking. They are also used to effectively control the release of a substance within the crosslinked structure. One example of controlled crosslinking is the addition of divalent calcium chloride to alginate. The mechanical properties of alginate hydrogel can be controlled with the crosslinking density, which is linked to the cation fraction. A disadvantage of using the aforementioned ionically crosslinked hydrogels is the dissolution of ions in the surrounding medium [26,27]. An advantage of hydrogels based on hydrophobic interactions is their ability to disperse any external forces, in particular through the micelles in the structure [28]. This peculiar ability relies on the molecular reversible association/dissociation of the hydrophobic moieties upon an external stress. An example of this could be a hydrogel made of acrylamide that acted as a hydrophilic domain and mixed with hydrophobic octyl phenol poly ethoxy ether. According to Liu et. al., this hydrogel showed great mechanical properties and attained self-healing properties [29].

The last type of physical crosslinking is the formation of hydrogels through crystallization. As the name suggests, the hydrogel is obtained through the formation of crystallites. The mechanical properties of this type of hydrogel can be changed by varying the concentration of an aqueous phase and the number of freeze-thawing cycles as well as the molecular weight. An example of this is the polyvinyl alcohol/alginate hydrogel, which was obtained from several freeze-thaw cycles. The crystallization commenced once the hydrogel was immersed into an aqueous NaCl solution, which increased its mechanical strength [30].

There are both advantages and disadvantages to the method of physical crosslinking. Compared to chemical crosslinking, hydrogels formed through physical crosslinking tend to lack mechanical strength and longevity. Physically crosslinked hydrogels are referred to as reversible hydrogels because most of the crosslinking mechanism can be reversed since no reactions take place in the formation of hydrogels. However, similar to chemical crosslinking, physical hydrogels tend to lack network homogeneity, which is expressed in the uneven distribution of swelling [31]. The advantage to physical crosslinking is the absence of any crosslinking agents that would otherwise be needed in covalently crosslinked hydrogels and which would need to be removed as they can be toxic and harmful [32].

### 2.2. Covalently Crosslinked Hydrogel

Compared to the physically crosslinked hydrogels, chemically crosslinked ones are not reversible and are in possession of a higher level of mechanical properties [26], such as the elastic modulus. This is achieved through the different methods of covalent crosslinking. Hydrogels can be crosslinked via thermal polymerization [33,34], photopolymerization [35,36,37], enzymatic crosslinking [38,39,40], and several other methods [32]. The photopolymerization crosslinking reaction involves a light source that initiates gel formation. The advantage of this method is region-selective crosslinking as it involves a photon-activated crosslinking of certain desired regions. Additionally, these reactions can undergo mild conditions and temperatures, which makes it a convenient method [4]. The downside to this method is the need for photosensitizers and prolonged exposure to irradiation. Different photosensitizers absorb photons of different wavelengths and hence need to be properly selected. There is also a risk of local temperature rise, which further increases toxicity levels. Generally, crosslinking through photopolymerization involves polymers with methacrylate groups, which allow for photoinitiation [41,42]. The double bond renders the compound highly sensitive to UV radiation and consequently to chain polymerization. One of the most researched photosynthesized hydrogels is methacrylated hyaluronic acid [43,44,45]. It serves well in biomedical applications and is synthesized through the UV radiation of methacrylated hyaluronic acid prepolymer solution in the presence of a photoinitiator.

The next method of crosslinking is through an enzymatic reaction. This method offers a stable and non-toxic method for producing hydrogels as the enzyme can have substrate specificity [46]. Polymer crosslinking can also occur in mild conditions such as atmospheric temperature and neutral pH. This allows for the usage of a wider range of polymers that are usually susceptible to harsh conditions. Additionally, enzymatically crosslinked hydrogels form strong covalent bonds and have a short gelation time. One of the examples of an enzyme used in enzymatic crosslinking is a transglutaminase used in the formation of a polyethylene glycol/hyaluronic acid hydrogel with hyaluronic acid (HA) used as a building block [47]. The formed hydrogel finds its use in regenerative medicine.

Crosslinked hydrogels can also be synthesized via through “click” chemistry. This entails different methods of polymer conjugation. These involve cycloaddition reactions such as the selective Diels-Alder reaction between a diene and a substituted alkene [48,49,50]. One of the advantages of this reaction is the absence of any initiators, but polymers have to be modified to incorporate a dienophile such as a furan functional group to ensure the desired reaction. An example of the Diels-Alder hydrogel is a hydrogel synthesized from hyaluronan methylfuran and bismaleimide polyethylene glycol. The formed hydrogel allows for the encapsulation of cells [51]. Another mechanism of click-chemistry is the copper catalyzed alkyne-azide cycloaddition reaction [52,53]. The benefits of this reaction method are the short gelation times and high product yields. However, on the other hand, the fast gelation results in a hydrogel with non-homogeneous viscosity. Another disadvantage of this method is the effectiveness of the copper catalyst, which is prone to oxidation and consequently to reduction in the reaction efficiency. To avoid problems with the copper catalyst, thiol-ene reaction can be employed. The reaction entails the use of thiol groups reacting with alkene groups [54,55,56]. In addition to the mentioned advantages of the catalyzed alkyne-azide reaction, thiol-ene based reactions lack oxygen sensitivity as well as any initiator compounds [57]. An example of this is a successfully photosynthesized polyethylene glycol hydrogel [58]. The hydrogel was synthesized from the chain-polymerization reaction of PEG diacrylate and lithium arylphosphate as a photoinitiator. The formed hydrogel can be used for cell encapsulation and tissue engineering as it offers good cytocompatibility.

Another method of synthesizing crosslinked hydrogels is through the formation of a Schiff base that is typically formed between amine and aldehyde groups [59,60,61]. The reaction between these groups forms an imine linkage that possesses an ability to uncouple and couple, creating self-healing characteristics [59,62]. An example is the hydrogel obtained from two naturally derived polymers: chitin and alginate. To successfully form a Schiff base, chitin had to be modified to obtain an acrylamide group and alginate to be oxidized. One of the important characteristics of the formed hydrogel is its inability to undergo self-healing in acidic conditions [61].

The next method of hydrogel formation is through the Michael addition [63,64]. The Michael reaction is an addition reaction between a nucleophile and substituted olefins. Usually, the electron-accepting compounds have electron withdrawing groups such as acrylate esters, maleimides, alkyl methacrylates, and acrylamides. The advantage of the Michael addition reaction is the mild reaction condition and high regioselectivity. An example of a Michael reaction hydrogel is a thiol-modified chitosan reacting with bismaleimide where an increase in the amount of bismaleimide resulted in the improvement of storage modulus values as the number of crosslinking points increased [65].

### 2.3. Interpenetrating Networks

There is a special class of chemically crosslinked hydrogels named interpenetrating networks (IPN) [66,67,68]. The presence of an IPN entails a crosslinking of two or more polymers, which can result in a new polymeric profile. To successfully synthesize a hydrogel with an interpenetrating network, the used polymers have to fulfil three criteria. First is that the used polymer must be synthesized or crosslinked in the presence of a second polymer. Second, the involved polymers should have similar kinetics. Lastly, there should be no phase separation in the hydrogel. IPN hydrogels can be synthesized from synthetic and natural polymers. Natural polymers consist of proteins and polysaccharides, whereas the synthetic polymers tend to have functional hydrophilic groups such as –COOH, amines, –OH, etc. A possible hydrogel with an IPN has been reported with poly(styrene-butadiene-styrene)/poly(N-isopropylacrylamide) (SBS/PNIPAM) [69]. The radical polymerization takes place in a benzene-tetrahydrofuran solvent mixture. The formed hydrogel has good elastic properties. IPN hydrogels synthesized with fibrous, stress-responsive polyisocyanide (PIC) and thermoresponsive PNIPAM have been reported, which can become up to 50 times stiffer upon external temperature changes.

### 2.4. Composite Hydrogels

Another type of hydrogel incorporates two different types of materials for the formation of a product with superior mechanical and biological properties. One of the main purposes of composite hydrogel formation is to synergistically combine the benefit of the separate constituents [70,71]. Generally, in the composite hydrogel, the organic phase acts as a hydrogel matrix, and the inorganic compound acts as a filler that imparts stiffness and rigidity. Composite hydrogels can be synthesized from chemically and physically crosslinked hydrogels. Most of the composite hydrogels are used in the cell encapsulation, which means that it is important to ensure an even rate of the degradation of organic and inorganic phases [72,73,74]. An advantage of a composite hydrogel is the increased mechanical properties [75]. A small nuance in the inorganic filling is the random dispersion in both the covalently and physically crosslinked hydrogels. An example of the composite hydrogel can be used as a mimic for the cartilage and to study the load bearing. A composite hydrogel was synthesized using polyvinyl alcohol and poly acrylic acid. The formed composite hydrogel successfully mimicked the behavior of cartilage, specifically an increase in the elastic pressure as the swelling increased [76].

## 3. Viscoelastic Behavior

**Figure 3:**
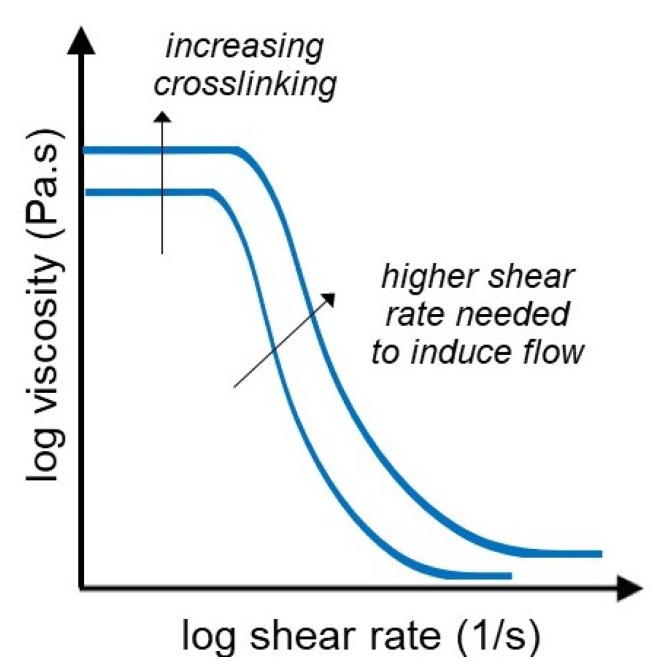
Schematic graph showing the flow behavior of hydrogels, represented by viscosity as a function of shear rate.

**Figure 4:**
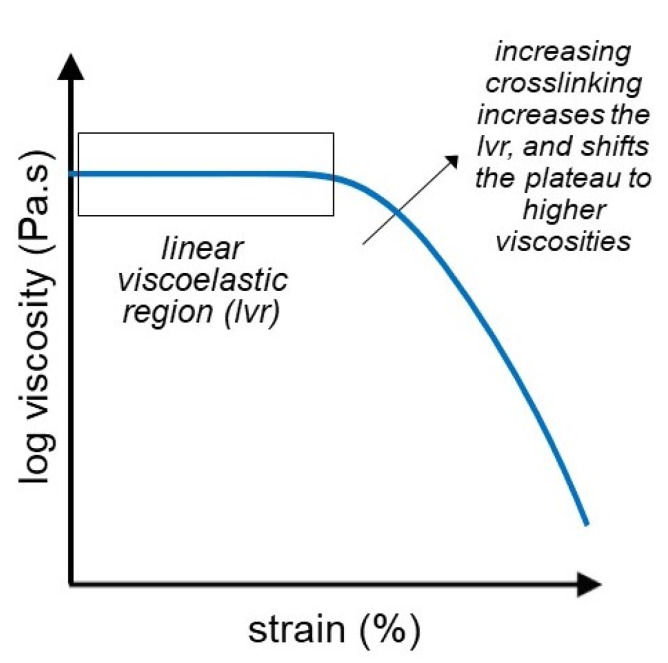
Strain sweep or amplitude sweep in hydrogels shows the linear viscoelastic region (LVR) as well as the threshold strain required.

**Figure 5:**
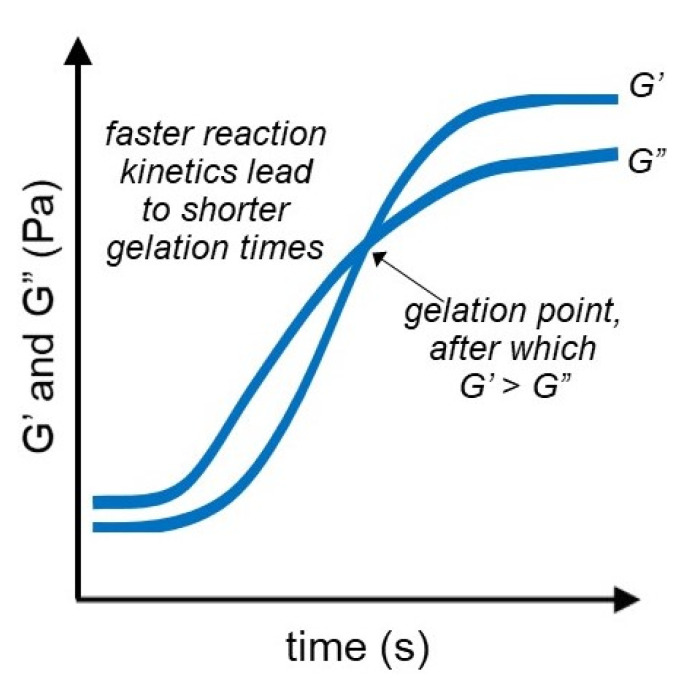
Time sweep experiment shows the formation of the network structure of the hydrogel over time. The crossover point of G’ and G” indicated the gelation point.

**Figure 6:**
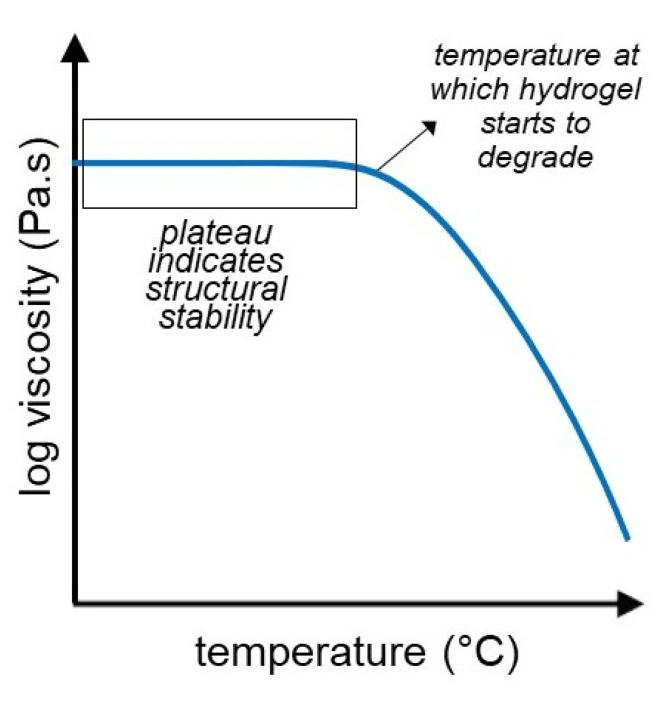
Temperature sweep experiments are a way to monitor the thermal stability of hydrogels. The point at which the viscosity drops suggests the temperature above which the hydrogel starts to degrade or de-crosslink.

**Figure 7:**
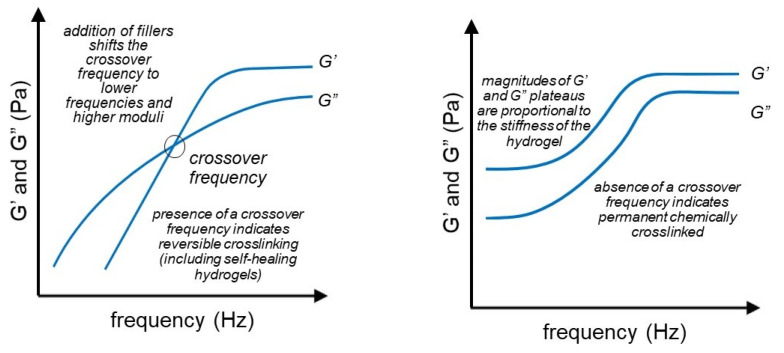
Frequency sweep experiments are a powerful tool in rheology of hydrogels which show the crosslinking behavior as well as the presence of a reversible network.

**Figure 8:**
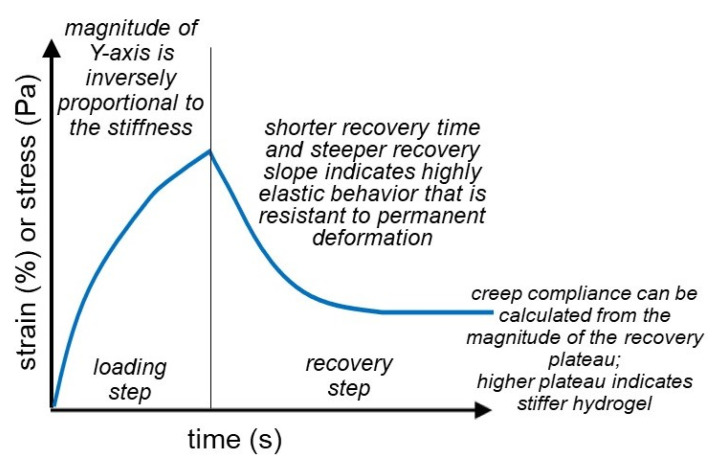
Creep recovery tests show the amount of applied strain a hydrogel can withstand and how the structure of the hydrogel recovers upon removal of this applied strain.

After understanding the different structures of hydrogel networks discussed in the previous section, in the following section we present the different rheological tests performed to characterize hydrogels. We discuss and evaluate each type of test with examples of how they are used. The results obtained from these rheological measurements will throw light upon the structure–property relationships of the hydrogels. For all these rheological experiments, schematic results are shown (Figure 3, Figure 4, Figure 5, Figure 6, Figure 7 and Figure 8) to enable easier interpretation of the results.

### 3.1. Flow Curves

Flow curves (steady shear flow) describe the rheological behavior of a material, more specifically the dependency of the viscosity on the applied shear rate. It is one of the most common characterization techniques as it is used for determining the tendency of a material to flow. Hydrogels are generally non-Newtonian fluids with shear-thinning behavior. Compared to Newtonian fluids, materials with shear-thinning behavior experience a decrease in the viscosity as the applied shear rate increases (as shown in Figure 3). This is an important trait of hydrogels that can be beneficial in their applications such as injection, drug delivery, and tissue engineering [77]. Gong et al. used flow curves to compare the viscosity of hydrogels with different crosslinking ratios [78]. In addition to studying the viscosity dependence on the shear rate of the hydrogels, Grela et al. used steady shear flow to study the thixotropy and its opposite phenomenon, rheopexy. Namely, after establishing that their hydrogels have a non-Newtonian shear thinning behavior, they determined that the viscosity of the hydrogel increases after every repeated shear flow test. As the shear rate increases, the viscosity of the hydrogel decreases up to a certain minimum. After this the shear rate is reduced, which leads to increased values in the viscosity, which are higher than the original viscosity values for the respective shear rate. This phenomenon is known as negative thixotropy or rheopexy [79].

### 3.2. Strain Sweep Test

Strain sweep test (amplitude sweep) is a common rheological test method employed to characterize hydrogels using increasing oscillatory strain at a constant frequency. The results of a strain sweep test are expressed via the storage(G’) and loss (G”)moduli of the hydrogel over an increasing strain range, which additionally gives insight into the Newtonian behavior or the linear viscoelastic region (LVR) of the material, as shown in Figure 4. The LVR of the hydrogels occurs at low shear stress, during which the moduli are independent of the increasing stress. Moreover, as the stress is increased the G’-G” crossover point potentially reaches the point at which the gel-sol transformation occurs, and the material starts to behave like a fluid [80]. The main purpose of strain sweep testing is to determine the linear viscoelastic region of the hydrogels.

Mendoza et al. performed strain sweep tests on cellulose nanofiber gels containing different wt.% fiber content. Using this characterization technique, they first determined the LVR region of the nanofiber gels, in which their elastic behavior (G’) predominates. Subsequently, they determined the crossover point at which the hydrogels converted to non-Newtonian shear-thinning fluids. Additionally, it was concluded that the increased wt.% of fiber content in the hydrogels led to a shorter LVR region and a distinct crossover point; the lowest amount of wt.% nanofiber was 0.09% gels, for which no crossover point was determined in the tested stress range [80]. In addition to determining the LVR and the crossover point of their PHEMA-based hydrogels, Nadgorny et al. used the strain sweep test to study the self-healing behavior of their gels. After the critical transition was achieved, the stress of the original LVR was decreased, at which point the hydrogels achieved 97% recovery of the initial G’ and G” moduli, hence demonstrating their self-healing behavior [81].

### 3.3. Time Sweep Test

Time sweep is a rheological technique frequently used for determining structural changes for a specific material over a certain period of time. Alterations in the structure such as the evaporation of solvent, curing, gelation, polymer degradation, or recovery directly impact the rheological properties of the compound, and such changes can be monitored via time sweep experiments. Time sweep experiments enable the study of the gelation process of the hydrogel as shown in Figure 5. The gelation time is defined as the crossover point of the storage (G’) and loss (G”) moduli. This crossover point is important because it indicates the kinetics of the gelation reaction. For instance, Deng et al. used oscillatory time strain to evaluate the dependency of storage modulus (G’) and loss modulus (G”) of HA/CMC hydrogels over time and determined the gelling time at the crossover point of the G’ and G” curves [82]. However, Balakrishnan et al. reported a limitation in this measurement because of the fast gelation of DDA-ChitHCl hydrogels—the gelation time could not be measured using oscillatory time sweep; nonetheless, the crossover point was still observed, and the storage modulus of the gel was higher than the loss modulus after gelling [83].

Studying the changes in the rheological behavior of the material over time also helps to evaluate the stability of the hydrogel. Bertasa et al. performed an oscillatory time sweep test for a time period of 60 min on two types of agar hydrogels in order to determine the stability of the samples; since there were no significant changes in the G’ and G” values over the tested time period, they concluded that the agar hydrogels have high stability, which can be important for some specific applications, making the time sweep test equally as important [84]. Self-healing behavior is analyzed similarly to evaluating hydrogel stability, by looking at the changes in the G’ and G” over time. Bian et al. used time sweep to test CPB-LNP0 and CPB-LNP4 hydrogels and determined that in 600 s, the G’ and G” values of the hydrogels recover to the original values, thus suggesting the complete healing and recovery of the hydrogel structure [85].

### 3.4. Temperature Sweep

The temperature sweep (temperature ramp) test is a rheological characterization technique that gives insight into the structure of the hydrogel when subject to a certain range of temperatures. This can be achieved by measuring the storage (G’) and loss (G”) moduli in a certain temperature range, indicating the structural stability of the hydrogel at different temperatures, along with the sol-gel transition of the hydrogel [86,87,88]. Antoniuk et al. performed a temperature sweep test on their samples of poly(N-isopropylacrylamide) microgel-based hydrogels to determine the temperature dependence of the hydrogel rheological properties in the range from 25 to 60 °C. They determined that both the storage and loss moduli decrease as the temperature increases. However, the slope of the storage modulus is steeper, which eventually leads to the two values crossing and the occurrence of the gel-sol transition. The crossover point is different for the hydrogels tested; namely, one of them is affected by the collapse in the microgel structure leading to a lower crossover point at T = 36 °C, whereas the other hydrogel is less affected, having a crossover point at a higher temperature of T = 41 °C. Subsequently, the samples were cooled down, and both G’ and G” were identical, thus demonstrating full reversibility [86].

Time sweeps can be used to monitor the thermal stability of hydrogels as shown in Figure 6. Garcia et al. used the temperature sweep test to evaluate the stability of xanthan gum-based hydrogels and its dependency on the montmorillonite reinforcement particles. Throughout the tested temperature range (25–90 °C) the storage and loss moduli had negative slopes for all hydrogels; however, in the first region the slope was gradual, whereas in the second region it was steep and more pronounced. The least stable hydrogel was the non-reinforced xanthan gum hydrogel, whereas the hydrogel with the highest concentration of montmorillonite particles was the most stable. Therefore, they concluded that MMT particles improve the stability and increase the critical temperature at which the hydrogels start to rapidly breakdown by improving the elasticity (G’) of the hydrogels at higher temperatures [87]. Farina et al. used the temperature sweep test to study chestnut starch hydrogels and their gelatinization behavior. Namely, as the temperature increases the G’ and G” values are stable; however, at 55 °C there is a positive slope, which is determined to be the onset gelatinization temperature. Subsequently, both G’ and G” curves have a very sharp increase, and both values achieve a maximum at T = 62.5 °C, which is the gelatinization peak of the hydrogel. At T = 65 °C, the G’ and G” values start to slowly decrease, which is the final gelatinization temperature when the starch gelatinization is complete. Compared to potato starch, the storage and loss moduli are almost constant after the gelatinization peak temperature, indicating that the chestnut starch is more stable. After gradual cooling, it was also determined that the G’ and G” values had marginally increased; therefore, it was concluded that the hydrogel is reinforced after cooling down to 25 °C [88].

### 3.5. Frequency Sweep

The frequency sweep test is another rheological method that determines the relationship between testing frequency and the storage (G’) and loss (G”) moduli of a material. Moreover, it gives insight into the viscoelastic properties and state of a material by comparing the two G’ and G” values over the frequency range [35,36]. Ajovalasit et al. used the frequency sweep test to evaluate the impact that additives have on the storage and loss moduli of a hydrogel over a given frequency range; namely, they concluded that all hydrogels have the properties of a viscoelastic liquid with positive slopes on the G’ and G”, with the loss modulus increasing faster. The addition of glycerol does not impact the rheological properties, whereas the addition of glutaraldehyde moves the G’ and G” crossover points to lower frequencies, while the addition of PVA decreases the storage and loss modulus values [89]. The different information that can be interpreted from frequency sweep rheograms is shown schematically in Figure 7. It should be noted that the scaling of the axes of the figures can be highly dependent on the stiffness and dynamics of the hydrogels.

Gu et al. compared the loss and storage moduli values of physically and hybrid chemically crosslinked hydrogels; the G’ and G” values of the physical hydrogels were highly frequency dependent with the storage modulus being significantly higher than the loss modulus at the highest frequencies. The hybrid hydrogels show similar properties to the chemically crosslinked hydrogels in that both have G’ and G” values independent of the frequency; the main difference between them is that chemically crosslinked hydrogels have a higher loss modulus, indicating less viscous flow behavior [90]. Basu et al. used frequency sweep to explore the structure of their ion-crosslinked nanocellulose hydrogels; namely, throughout the frequency range, the storage modulus was larger than the loss modulus, which shows the solid-like structure of the hydrogel. Moreover, no crossover point was observed between the storage and loss modulus for the tested frequencies, demonstrating that the tested hydrogels possess entangled fibrous networks [91]. Finally, Jamburidze et al. used frequency sweep tests to compare their agarose hydrogel and biological tissues. The G’/G” ratio of the tested hydrogels was determined to be 10, which is similar to the properties reported for naturally occurring tissues, thus making these hydrogels potentially viable in tissue engineering applications [92].

### 3.6. Creep Compliance, Creep Recovery, and Stress Relaxation

The creep test is a rheological method that describes the tolerance of a material that deforms after a constant static load is applied, an occurrence also known as compliance. Namely, a constant stress is applied to a sample, which leads to an increasing strain that reaches an equilibrium after a certain time. At this point, no further stress is applied, and the recovery of the sample is recorded over a certain fixed time, as shown in Figure 8. In addition, these creep recovery tests can be repeated multiple times in succession, which can provide insight into the behavior of the material after an approximation of real use. Farell et al. used creep compliance tests to compare the compliance of pure and blended hydrogels in relation to their composition. Pure collagen hydrogels were most susceptible to deformation, followed by pure HA hydrogels, with the blended hydrogels being the most resistant [93]. In addition to using creep compliance tests to compare hydrogels with different montmorillonite loadings to determine the tolerance of deformation, Garcia et al. used the creep recovery test to further compare the mean retardation time constant and the recovery percentage with the MMT loading. They observed that the MMT particles improved the elastic behavior of the hydrogels first by decreasing the compliance and thus increasing the elasticity, and second by reducing the mean retardation time constant.

Stress relaxation is a rheological test that can be considered the inverse of the creep compliance test. Instead of applying a constant stress and measuring the strain that the sample is experiencing, a stress relaxation test subjects the sample to a constant strain and measures the stress exerted by the sample. It gives insight into how well materials can dissipate the stress over time at a constant strain. Hashemnejad et al. performed a stress relaxation test on their molecular gels for a certain time range, after which they observed a decreased stress applied by the hydrogel. Specifically, the stress had reduced to around 50% of the initially exerted stress in the same time span of 30 min [94]. Farell et al. supplemented their creep recovery tests with a stress relaxation test in order to compare the relaxation time of the pure and blended hydrogels. All of the hydrogels exhibited non-linear time response, establishing their viscoelastic nature. Pure collagen hydrogels had a relaxation time of 40 s, whereas one of HA hydrogels was 120 s. Moreover, blended hydrogels had a similar relaxation time to the collagen-based one. This demonstrates the ability of the collagen and blended hydrogels to relieve the stress sooner than HA hydrogels, which could potentially be important in future applications [93].

## 4. Product Design as Related to Applications 

In product design steps the two factors (chemical structure and mechanical properties) elucidated in the two sections above are both used in order to finely tune a given product for specific applications. In this paragraph we provide relevant examples.

### 4.1. Drug Delivery

As explained previously, hydrogels are insoluble crosslinked structures that can contain high volumes of water and have an elastic structure. Due to their compatibility with aqueous environments, hydrogels are an attractive material in biological applications, specifically in drug delivery. The different methods of direct drug delivery include environment-responsive drug release, diffusion release, structure degradation, swelling, and chemical mechanism [95]. Each of the methods of release have distinguishing features and advantages as well as disadvantages. In case of the drug delivery by environmental stimuli, hydrogels react to physical stimuli such as temperature, light, or pressure, and chemical stimuli such as pH, or the ionic strength of the surroundings. Under these conditions, the hydrogel structure would swell, and the polymer structure would stretch and release a drug [96].

In the case of drug release by diffusion, there are two types of hydrogel/drug structures that ensure that mechanism. The first entails an encapsulation of the drug in the layer of hydrogel where the drug is released at a constant rate. Extra care and design considerations are of importance as the polymeric layer can rupture and release the drug at an undesired rate. The second one is the diffusion of the drug into a hydrogel, forming a matrix, and the diffusion rate is dependent on time. The diffusion starts once the system comes into contact with the aqueous surrounding and swells, stretching the polymer structure [97]. Chemical mechanism entails structural degradation as the polymeric layer erodes upon the arrival at the targeted area, and the drug within the system is released. Out of the mentioned methods of drug delivery, stimuli-responsive release is the optimal method as it offers a controlled drug delivery and can avoid non-targeted areas.

An understanding of the swelling mechanism can help control the desired drug delivery; to successfully assess the hydrogel for biocompatibility, several aspects have to be considered. In addition to the previously mentioned environmental influence, an interaction between a drug of choice and hydrogel has to be considered. Those could be physical interactions or covalent interactions; there is also a surface diffusion control that can be applied to the hydrogel that could facilitate the release of a drug at a specific temperature or pH. In the following section, we describe many different types of hydrogel compositions and their utilization in drug delivery.

Chen et al. evaluated hydrogels as injectable drug delivery systems. They concluded that lower loss (G”) and storage (G’) moduli along with lower viscosity improve the injectability of a hydrogel; the frequency and strain sweep indicate that the 5% hydrogels have lower yield strain and flow more easily compared to 7.5% hydrogels, thus potentially making them a more viable option for injectability. Shear thinning is another property that is important to evaluate the injectability of a hydrogel because, upon the application of strain, the hydrogel should have a lower viscosity, thereby making it easier to inject; continuous flow experiments indicate that both 5% and 7.5% hydrogels manifest a shear-thinning property. Finally, cyclic strain sweep tests indicate the recoverability of both hydrogels as both display high decreases in the loss and storage moduli upon the application of high strains; however, at low strain, both storage and loss moduli return to their original values. Moreover, the 5% hydrogel also exhibited shear banding, which is a very high drop in shear stress at given shear rates, which is also a positive property for injectability applications. Overall, both hydrogels demonstrate shear-thinning abilities and a change in loss and storage modulus at different strain; however, the 5% hydrogel has overall lower viscosity, storage, and loss moduli compared to the 7.5% hydrogel, which leads to a conclusion that it should be more suited and easier to inject [98].

Another notable example can be found in research carried out by Deng et al., who developed various injectable in situ cross-linking hyaluronic acid/carboxymethyl cellulose hydrogels and evaluated their injectability. Rheological evaluation of the three different hydrogels concluded that all samples have similar properties and demonstrate shear-thinning behavior; however, the sample that exhibited the shortest gelation time, higher storage, and loss moduli compared to the other two hydrogels but also more significant shear thinning, making it the more suitable hydrogel for injectable drug delivery [82]. Afzal et al. developed alginate/chitosan hydrogels designed for drug encapsulation. They reported that rheological measurements at neutral pH resulted in a reduced viscosity and an increased elasticity of the composite hydrogels compared to pure chitosan hydrogels, thereby making this type of hydrogel suitable for drug encapsulation and drug delivery [99].

### 4.2. Wound Dressing/Tissue Engineering

Skin is one of the largest human organs and has a wide array of functions. One of the main functions is body protection, moisture level stability, and consequential ability to self-regenerate. The ability to self-regenerate or heal depends on the type of wound and severity as well as one’s intrinsic ability to undergo self-regeneration. In general, there are two types of wounds: acute and chronic [100]. Acute wounds are further subdivided into physical, chemical, and thermal wounds, whereas chronic wounds are usually caused by underlying physiological bodily issues such as diabetes or angiogenesis and generally require a more intensive care. Some of the chronic wounds tend to not heal completely, which reduces the quality of life and increases the risk of further infection and hence calls for the reduction of unnecessary exposure to the outside. In either case, it would be helpful and beneficial to use an external layer of material that could either speed up the healing process or protect the wound from external risks. Many traditional options are currently being used to treat and heal open wounds, but among the new advancements, hydrogels have potential. The water retention capacity of hydrogels makes them a perfect candidate for wound dressing as they can hold in optimal moisture levels within the target area [101]. In addition to that, if the hydrogels are made from natural polymers, they would more likely be biocompatible and thus suitable for this application [102].

Morariu et al. developed polyvinyl alcohol (PVA) hydrogels, which include a magnetic particle as filler, for which they highlighted several necessary properties they needed in order to be applicable to wound dressing, such swelling ability, surface roughness, transparency, and viscoelastic behavior and flexibility. Using rheological testing, they compared several different hydrogels compositions; namely hydrogels with the lowest or the highest glutaraldehyde cross linker quantity possess the best viscoelastic properties, for example, higher G’ and lower G” moduli. Furthermore, filler incorporation in the hydrogels decreases the G’ modulus for any glutaraldehyde concentration. However, filler addition improves the surface roughness along with the swelling abilities, which are already the most promising at the lowest glutaraldehyde quantity, thus making the filler incorporated-low glutaraldehyde hydrogels the best performing hydrogels in wound dressing application [103].

Similarly, Yin et al. compared the moduli of different hydrogels made of gelatin (GA), hyaluronic acid (HA), and cellulose nanocrystals (CNC), using dynamic mechanical analysis. While the loss modulus was not impacted by the different composition of the hydrogels, the elastic storage modulus was increased by the incorporation of CNC, giving the GA-HA-CNC hydrogels the best viscoelastic properties; thus, they are more likely to be applied as wound dressing material than the other hydrogels tested [104]. Finally, Quah et al. used rheological characterization to compare the ability of the developed hydrogel to transition from weak to stiff gels. Controlling the transition temperature by changing the hydrogels content could be beneficial for the wound application of the gels, specifically by having a weak gel at a low temperature that is easy to apply that would subsequently transition into a stiff gel when reaching temperatures close to body temp [105].

Along with the ability to help regenerate the wounded areas of the skin, hydrogels have also gained significant attention regarding their application tissue engineering with their highly hydrophobic, high water content, three-dimensional structure. As there are more options as well as demand for organ transplants, there are also likely to be more challenges and considerations that would require a strategic approach. Typically, in tissue engineering, cells of a patient are incorporated into a hydrogel polymer scaffold. Different methods of tissue engineering are explored nowadays, and they can employ either natural or synthetic hydrogels. Another important factor considered is that the hydrogels used must have high biodegradability outside and inside the body and can be easily incorporated by the original host tissue. In this case, natural polymers have an advantage as they have a higher biocompatibility. One example of a biocompatible hydrogel is made from an alginate molecule that has a sufficient amount of hydroxyl groups, making its derivatives hydrophilic. Another natural hydrogel is made from collagen or silk, which can be degraded in the body of a patient using specific enzymes which can be added post procedure to safely remove excess hydrogels. Additionally, it is important to ensure appropriate mechanical properties for the hydrogel to create and maintain a structured scaffold with spacing for the unhindered cell growth/tissue development. In this regard, natural polymers tend to lack strength compared to their synthetic counterparts. With tissue engineering, hydrogel scaffolds can be applied in three different ways: for space filling agents, bio-active molecule delivery, and cells or tissue delivery [106]. With the space filling agents, the polymer scaffold acts as a new growth platform for the cells as it provides the optimal growth medium with the controlled diffusion of different nutrients and metabolites. For this, the 3D scaffold structure of the hydrogel ensures an optimal growth. In case of molecule delivery, it is a non-area-specific delivery of drugs into a patient, meaning that larger doses are required. In other case scenarios, injectable hydrogels can be used for soft and hard tissues. Hydrogels can be applied in cardiac-related operations to deliver viable cells and molecules [107]. Additionally, for hard tissues such as bones, injectable hydrogels act as a delivery method rather than the load-bearing material since it is still a challenge to raise the mechanical properties to bear the loads.

In relation to tissue engineering applications, researchers have used rheological characterization in several ways. Alarke et al. and Alinejad et al. studied the gelation time in order to understand the injectability behavior of the hydrogels and evaluate the hydrogels most suitable for injection and use in tissue engineering. Another parameter that was researched was the self-healing behavior of given hydrogels using an alternating step-strain sweep; they established that the tested hydrogels have excellent recoverability and hence they would be suitable for tissue engineering applications [108,109]. Finally, Dorishety et al. used rheological tests to compare the viscoelastic properties of given hydrogels to biological tissues; specifically, they concluded the storage modulus of regenerated silk fibroin (RSF)/nanocellulose composite hydrogels is close to the one of articular cartilage tissue and that compression modulus of the RSF/nanocellulose hydrogels was on par with human medial meniscus tissue [110]. Katoch et al. compared the viscoelastic properties of hydrogels to muscle, bone, and cartilage tissue and concluded that hydrogels, depending on the composition, can mimic all three different tissues and thus could be significant for tissue engineering [111].

### 4.3. Food 

Hydrogels have also found their place in the food industry, specifically food packaging. Their high water content, three-dimensional structure allows for a biocompatible relationship with edible products. Additionally, hydrogels can be made from natural polymers, which increases their biocompatibility and biodegradability. The natural polymers include proteins and polysaccharides such as chitin, chitosan, hyaluronic acid, and cellulose derivatives. Their ability to take up water as much as 100% of their weight allows them to be employed for humidity control in food packaging. Humidity control could help preserve food freshness for longer periods of time as the appearance of mold and bacteria is avoided. It could also help preserve the state of dry packaged foods. For these kinds of specializations, hydrogels’ properties can be tinkered with, with changes to the crosslinking density, polymer type, reaction conditions, and the pH. Along with the swelling and water intake abilities, hydrogels have a corresponding application as mechanical properties.

Gong et al. developed thermosensitive hydrogels suitable for use in the food industry. Using rheology, they observed that the non-Newtonian, shear-thinning nature of the hydrogels could improve food texture and food processability. Moreover, depending on the hydrogel composition, they could tune the viscosity as well as the sol-gel transition temperature [78]. The temperature sensitive behavior of the hydrogels is demonstrated by a temperature sweep that showed all hydrogels exhibiting increasing complex viscosity and storage modulus with decreasing of the temperatures, with the most prominent change happening between 35 and 25 °C [78]. This could present a significant advantage for these hydrogels in the food industry because of the different temperatures employed in formation and processing of food.

### 4.4. Agriculture 

Hydrogels have been used in agriculture for the absorption of unwanted chemicals in an attempt to clean water as a result of high pollution in the areas of pharmaceutical, petroleum, fertilizers, and dyes. One of the researched targeted hazardous pollutants is heavy metals. A particular compound of interest is hexavalent chromium, which is considered as a heavy toxic metal and is deemed to be a problem by previously mentioned sources. Rizzo et al. synthesized carbohydrate-derived gels capable of absorbing heavy metals from wastewater. Using rheology as one of the characterization methods for the hydrogels, they concluded that gels with tunable strength could be synthesized [112]. 

A significant amount of research has been conducted on the usage of hydrogels in biomedical fields, but the focus is also shifting towards other industries. The versatile nature of the crosslinking methods of hydrogels allows them to expand their use. Additionally, the use of natural polymers renders it a viable alternative in food packaging, personal care, antibacterial substances, membranes, sensors, and many other potential applications [113,114,115,116].

## 5. Future Perspectives and Conclusions

Hydrogels offer a wide range of applications due to their various crosslinking methods and the corresponding mechanical properties. Ranging from the food packaging to tissue engineering, hydrogels are gaining more attention and offer a great substitute for existing materials. The crosslinking methods include physical crosslinking, covalent crosslinking, interpenetrating crosslinking, and composite hydrogels. The physical crosslinking includes crosslinking by entanglement, through hydrogen bonding, and ionic interactions. Covalently crosslinked hydrogels can be formed through photopolymerization, through different reactions of click chemistry and using enzymes. Using various tests, rheological properties of the hydrogels such as gelation time, storage and loss modulus, and self-healing behavior can be established, all of which contribute towards evaluating the given hydrogel for the intended application. Drug delivery hydrogels have various methods for direct drug delivery, which include environment-responsive drug release, diffusion release, structure degradation, swelling, and chemical mechanism. For instance, in order to be applied as injectable drug delivery systems, most importantly hydrogels need to be biocompatible and possess rheological properties such as low viscosity, storage, and loss moduli as well as a short gelation time. Similarly, biocompatible hydrogels with high swelling capabilities offer optimal moisture levels, thus making them ideal for wound dressing applications.

Along with the aforementioned applications, hydrogels are in tissue engineering applications due to their tunable mechanical properties. The ability to take impact could be beneficial for cartilage replacement, heart tissue reparations, and wound healing. The primary caveat is to ensure the purity of the formed hydrogels, which results in the use of natural polymers in the formation of hydrogels. Finally, the innate biocompatible and biodegradability of hydrogels made from natural polymers, along with their ability to absorb high amounts of water, makes them very suitable for use in the food industry, specifically as food packaging. Considering all the different types of hydrogels and the various properties they possess, in time the number of relevant applications of hydrogels will grow, as they show very high potential that needs more research to be properly utilized.

On a general level the interrelation between chemical structure and mechanical behavior constitutes an essential tool when aiming at designing specific kind of hydrogels for a given application. This interdisciplinary approach is characterized by relevant level of difficulties related to complex nature of hydrogels. This complexity stems from molecular factors, the number of components present, and the kind of crosslinking. However, major challenges arise also when translating these molecular characteristics to the macromolecular level. For example, the molecular reversibility of hydrogen bonds used for crosslinking results in (segmental) chain mobility effects that crucially determine the mechanical behavior. It is worth noting here that three different length scales are involved in this translation: molecular, macromolecular, and macroscopic. Unveiling such interrelationships (at the different length scales) through rheology and their use is then paramount for the product designer in order for material selection in several different application areas.

## References

[1] Nishinari K. (2005). Study on hydrogels in food physics. J. Jpn. Soc. Food Sci. Technol..

[2] Yan C., Pochan D.J. (2010). Rheological properties of peptide-based hydrogels for biomedical and other applications. Chem. Soc. Rev..

[3] Mondal S., Das S., Nandi A.K. (2020). A review on recent advances in polymer and peptide hydrogels. Soft Matter.

[4] Hu W., Wang Z., Xiao Y., Zhang S., Wang J. (2019). Advances in crosslinking strategies of biomedical hydrogels. Biomater. Sci..

[5] Zhai M., Xu Y., Zhou B., Jing W. (2018). Keratin-chitosan/n-ZnO nanocomposite hydrogel for antimicrobial treatment of burn wound healing: Characterization and biomedical application. J. Photochem. Photobiol. B Biol..

[6] Deng Y., Huang M., Sun D., Hou Y., Li Y., Dong T., Wang X., Zhang L., Yang W. (2018). Dual physically cross-linked κ-carrageenan-based double network hydrogels with superior self-healing performance for biomedical application. ACS Appl. Mater. Interfaces.

[7] Li Y., Neoh K.G., Kang E.-T. (2004). Poly(vinyl alcohol) hydrogel fixation on poly(ethylene terephthalate) surface for biomedical application. Polymer.

[8] Abdel-Mohsen A.M., Aly A.S., Hrdina R., Montaser A., Hebeish A. (2011). Eco-synthesis of PVA/chitosan hydrogels for biomedical application. J. Polym. Environ..

[9] Shao C., Chang H., Wang M., Xu F., Yang J. (2017). High-strength, tough, and self-healing nanocomposite physical hydrogels based on the synergistic effects of dynamic hydrogen bond and dual coordination bonds. ACS Appl. Mater. Interfaces.

[10] Wang Y.J., Zhang X.N., Song Y., Zhao Y., Chen L., Su F., Li L., Wu Z.L., Zheng Q. (2019). Ultrastiff and Tough Supramolecular Hydrogels with a dense and robust hydrogen bond network. Chem. Mater..

[11] Tong X., Du L., Xu Q. (2018). Tough, adhesive and self-healing conductive 3D network hydrogel of physically linked functionalized-boron nitride/clay /poly(N-isopropylacrylamide). J. Mater. Chem. A.

[12] Liang Y., Xue J., Du B., Nie J. (2019). Ultrastiff, tough, and healable ionic–hydrogen bond cross-linked hydrogels and their uses as building blocks to construct complex hydrogel structures. ACS Appl. Mater. Interfaces.

[13] Lin Y., Zhang H., Liao H., Zhao Y., Li K. (2019). A physically crosslinked, self-healing hydrogel electrolyte for nano-wire PANI flexible supercapacitors. Chem. Eng. J..

[14] Liu S., Oderinde O.K., Hussain I., Yao F., Fu G. (2018). Dual ionic cross-linked double network hydrogel with self-healing, conductive, and force sensitive properties. Polymer.

[15] Deng Y., Hussain I., Kang M., Li K., Yao F., Liu S., Fu G. (2018). Self-recoverable and mechanical-reinforced hydrogel based on hydrophobic interaction with self-healable and conductive properties. Chem. Eng. J..

[16] Zhang X.N., Wang Y.J., Sun S., Hou L., Wu P., Wu Z.L., Zheng Q. (2018). A tough and stiff hydrogel with tunable water content and mechanical properties based on the synergistic effect of hydrogen bonding and hydrophobic interaction. Macromolecules.

[17] Chang X., Geng Y., Cao H., Zhou J., Tian Y., Shan G., Bao Y., Wu Z.L., Pan P. (2018). Dual-Crosslink Physical Hydrogels with High Toughness Based on Synergistic Hydrogen Bonding and Hydrophobic Interactions. Macromol. Rapid. Commun..

[18] Mihajlovic M., Staropoli M., Appavou M.-S., Wyss H.M., Pyckhout-Hintzen W., Sijbesma R.P. (2017). Tough supramolecular hydrogel based on strong hydrophobic interactions in a multiblock segmented copolymer. Macromolecules.

[19] Löwenberg C., Balk M., Wischke C., Behl M., Lendlein A. (2017). Shape-memory hydrogels: Evolution of structural principles to enable shape switching of hydrophilic polymer Networks. Acc. Chem. Res..

[20] Mredha M.T.I., Pathak S.K., Tran V.T., Cui J., Jeon I. (2019). Hydrogels with superior mechanical properties from the synergistic effect in hydrophobic–hydrophilic copolymers. Chem. Eng. J..

[21] Oveissi F., Naficy S., Le T.Y.L., Fletcher D.F., Dehghani F. (2018). Tough and processable hydrogels based on lignin and hydrophilic polyurethane. ACS Appl. Bio Mater..

[22] George J., Hsu C.C., Nguyen L.T.B., Ye H., Cui Z. (2019). Neural tissue engineering with structured hydrogels in CNS models and therapies. Biotechnol. Adv..

[23] Zhao D., Feng M., Zhang L., He B., Chen X., Sun J. (2021). Facile synthesis of self-healing and layered sodium alginate/polyacrylamide hydrogel promoted by dynamic hydrogen bond. Carbohydr. Polym..

[24] Guo Y., Zhou X., Tang Q., Bao H., Wang G., Saha P. (2016). A self-healable and easily recyclable supramolecular hydrogel electrolyte for flexible supercapacitors. J. Mater. Chem. A.

[25] Zhang M., Yin Q., Ji X., Wang F., Gao X., Zhao M. (2020). High and fast adsorption of Cd(II) and Pb(II) ions from aqueous solutions by a waste biomass based hydrogel. Sci. Rep..

[26] Chang B., Ahuja N., Ma C., Liu X. (2017). Injectable scaffolds: Preparation and application in dental and craniofacial regeneration. Mater. Sci. Eng. R Rep..

[27] Jeon O., Alt D.S., Ahmed S.M., Alsberg E. (2012). The effect of oxidation on the degradation of photocrosslinkable alginate hydrogels. Biomaterials.

[28] Jiang H., Duan L., Ren X., Gao G. (2019). Hydrophobic association hydrogels with excellent mechanical and self-healing properties. Eur. Polym. J..

[29] Jiang G., Liu C., Liu X., Zhang G., Yang M., Liu F. (2009). Construction and properties of hydrophobic association hydrogels with high mechanical strength and reforming capability. Macromol. Mater. Eng..

[30] Jiang X., Xiang N., Zhang H., Sun Y., Lin Z., Hou L. (2018). Preparation and characterization of poly (vinyl alcohol)/sodium alginate hydrogel with high toughness and electric conductivity. Carbohydr. Polym..

[31] Hoffman A. (2012). Hydrogels for biomedical applications. Adv. Drug Deliv. Rev..

[32] Hennink W.E., van Nostrum C.F. (2012). Novel crosslinking methods to design hydrogels. Adv. Drug Deliv. Rev..

[33] Wang H., Zhu D., Paul A., Cai L., Enejder A., Yang F., Heilshorn S.C. (2017). Covalently Adaptable Elastin-Like Protein–Hyaluronic Acid (ELP–HA) Hybrid hydrogels with secondary thermoresponsive crosslinking for injectable stem cell delivery. Adv. Funct. Mater..

[34] Shin J.Y., Yeo Y.H., Jeong J.E., Park S.A., Park W.H. (2020). Dual-crosslinked methylcellulose hydrogels for 3D bioprinting applications. Carbohydr. Polym..

[35] Zhou Y., Zhao S., Zhang C., Liang K., Li J., Yang H., Gu S., Bai Z., Ye D., Xu W. (2018). Photopolymerized maleilated chitosan/thiol-terminated poly (vinyl alcohol) hydrogels as potential tissue engineering scaffolds. Carbohydr. Polym..

[36] Brown T.E., Carberry B.J., Worrell B.T., Dudaryeva O.Y., McBride M.K., Bowman C.N., Anseth K.S. (2018). Photopolymerized dynamic hydrogels with tunable viscoelastic properties through thioester exchange. Biomaterials.

[37] Zhou Y., Liang K., Zhao S., Zhang C., Li J., Yang H., Liu X., Yin X., Chen D., Xu W. (2018). Photopolymerized maleilated chitosan/methacrylated silk fibroin micro/nanocomposite hydrogels as potential scaffolds for cartilage tissue engineering. Int. J. Biol. Macromol..

[38] Zhong Y., Wang J., Yuan Z., Wang Y., Xi Z., Li L., Liu Z., Guo X. (2019). A mussel-inspired carboxymethyl cellulose hydrogel with enhanced adhesiveness through enzymatic crosslinking. Colloids Surf. B Biointerfaces.

[39] Kim S.-H., Kim K., Kim B.S., An Y.-H., Lee U.-J., Lee S.-H., Kim S.L., Kim B.-G., Hwang N.S. (2020). Fabrication of polyphenol-incorporated anti-inflammatory hydrogel via high-affinity enzymatic crosslinking for wet tissue adhesion. Biomaterials.

[40] Wei Q., Duan J., Ma G., Zhang W., Wang Q., Hu Z. (2019). Enzymatic crosslinking to fabricate antioxidant peptide-based supramolecular hydrogel for improving cutaneous wound healing. J. Mater. Chem. B.

[41] Wang Y., Ma M., Wang J., Zhang W., Lu W., Gao Y., Zhang B., Guo Y. (2018). Development of a photo-crosslinking, biodegradable GelMA/PEGDA hydrogel for guided bone regeneration materials. Materials.

[42] Kim H., Kim J., Choi J., Park Y., Ki C. (2018). Characterization of silk hydrogel formed with hydrolyzed silk fibroin-methacrylate via photopolymerization. Polymer.

[43] Poldervaart M.T., Goversen B., de Ruijter M., Abbadessa A., Melchels F.P., Öner F.C., Dhert W.J., Vermonden T., Alblas J. (2017). 3D bioprinting of methacrylated hyaluronic acid (MeHA) hydrogel with intrinsic osteogenicity. PLoS ONE.

[44] Spearman B.S., Agrawal N.K., Rubiano A., Simmons C.S., Mobini S., Schmidt C.E. (2020). Tunable methacrylated hyaluronic acid-based hydrogels as scaffolds for soft tissue engineering applications. J. Biomed. Mater. Res. Part A.

[45] Xiao W., Qu X., Li J., Chen L., Tan Y., Li K., Li B., Liao X. (2019). Synthesis and characterization of cell-laden double-network hydrogels based on silk fibroin and methacrylated hyaluronic acid. Eur. Polym. J..

[46] Teixeira L.M., Feijen J., van Blitterswijk C., Dijkstra P.J., Karperien M. (2012). Enzyme-catalyzed crosslinkable hydrogels: Emerging strategies for tissue engineering. Biomaterials.

[47] Ranga A., Lutolf M.P., Hilborn J., Ossipov D.A. (2016). Hyaluronic acid hydrogels formed in situ by transglutaminase-catalyzed reaction. Biomacromolecules.

[48] Bi B., Ma M., Lv S., Zhuo R., Jiang X. (2019). In-situ forming thermosensitive hydroxypropyl chitin-based hydrogel crosslinked by Diels-Alder reaction for three dimensional cell culture. Carbohydr. Polym..

[49] Li S., Wang L., Yu X., Wang C., Wang Z. (2018). Synthesis and characterization of a novel double cross-linked hydrogel based on Diels-Alder click reaction and coordination bonding. Mater. Sci. Eng. C.

[50] Bai X., Lü S., Cao Z., Ni B., Wang X., Ning P., Ma D., Wei H., Liu M. (2017). Dual crosslinked chondroitin sulfate injectable hydrogel formed via continuous Diels-Alder (DA) click chemistry for bone repair. Carbohydr. Polym..

[51] Smith L.J., Taimoory S.M., Tam R.Y., Baker A.E.G., Mohammad N.B., Trant J.F., Shoichet M.S. (2018). Diels–alder click-cross-linked hydrogels with increased reactivity enable 3D cell encapsulation. Biomacromolecules.

[52] Hodgson S.M., McNelles S.A., Abdullahu L., Marozas I.A., Anseth K.S., Adronov A. (2017). Reproducible dendronized PEG hydrogels via SPAAC cross-linking. Biomacromolecules.

[53] Liu X., Miller A.L., Xu H., Waletzki B.E., Lu L. (2019). Injectable Catalyst-Free Poly(Propylene Fumarate) System Cross-Linked by Strain Promoted Alkyne–Azide Cycloaddition Click Chemistry for Spine Defect Filling. Biomacromolecules.

[54] Michel S.E., Dutertre F., Denbow M.L., Galan M.C., Briscoe W.H. (2019). Facile synthesis of chitosan-based hydrogels and microgels through thiol–ene photoclick cross-linking. ACS Appl. Bio Mater..

[55] McOscar T.V.C., Gramlich W.M. (2018). Hydrogels from norbornene-functionalized carboxymethyl cellulose using a UV-initiated thiol-ene click reaction. Cellulose.

[56] Lee H.J., Fernandes-Cunha G.M., Myung D. (2018). In situ-forming hyaluronic acid hydrogel through visible light-induced thiol-ene reaction. React. Funct. Polym..

[57] Gopinathan J., Noh I. (2018). Click chemistry-based injectable hydrogels and bioprinting inks for tissue engineering applications. Tissue Eng. Regen. Med..

[58] Lin C.-C., Raza A., Shih H. (2011). PEG hydrogels formed by thiol-ene photo-click chemistry and their effect on the formation and recovery of insulin-secreting cell spheroids. Biomaterials.

[59] Vahedi M., Barzin J., Shokrolahi F., Shokrollahi P. (2018). Self-Healing, Injectable Gelatin Hydrogels Cross-Linked by Dynamic Schiff Base Linkages Support Cell Adhesion and Sustained Release of Antibacterial Drugs. Macromol. Mater. Eng..

[60] Ding X., Li G., Xiao C., Chen X. (2019). Enhancing the stability of hydrogels by doubling the schiff base linkages. Macromol. Chem. Phys..

[61] Jalalvandi E., Hanton L.R., Moratti S.C. (2017). Schiff-base based hydrogels as degradable platforms for hydrophobic drug delivery. Eur. Polym. J..

[62] Li S., Pei M., Wan T., Yang H., Gu S., Tao Y., Liu X., Zhou Y., Xu W., Xiao P. (2020). Self-healing hyaluronic acid hydrogels based on dynamic Schiff base linkages as biomaterials. Carbohydr. Polym..

[63] Pupkaite J., Rosenquist J., Hilborn J., Samanta A. (2019). Injectable shape-holding collagen hydrogel for cell encapsulation and delivery cross-linked using thiol-michael addition click reaction. Biomacromolecules.

[64] Nonsuwan P., Matsugami A., Hayashi F., Hyon S.-H., Matsumura K. (2019). Controlling the degradation of an oxidized dextran-based hydrogel independent of the mechanical properties. Carbohydr. Polym..

[65] Guaresti O., Basasoro S., Gonzalez K., Eceiza A., Gabilondo N. (2019). In situ cross–linked chitosan hydrogels via Michael addition reaction based on water–soluble thiol–maleimide precursors. Eur. Polym. J..

[66] Parhi R. (2017). Cross-linked hydrogel for pharmaceutical applications: A review. Adv. Pharm. Bull..

[67] Zhu T., Cheng Y., Cao C., Mao J., Li L., Huang J., Gao S., Dong X., Chen Z., Lai Y. (2020). A semi-interpenetrating network ionic hydrogel for strain sensing with high sensitivity, large strain range, and stable cycle performance. Chem. Eng. J..

[68] Feig V.R., Tran H., Lee M., Bao Z. (2018). Mechanically tunable conductive interpenetrating network hydrogels that mimic the elastic moduli of biological tissue. Nat. Commun..

[69] Liu X., Zhang A., Su G., Xie H., Shi X., Li L. (2019). Preparation and Characterization of Temperature-Sensitive Poly (Styrene-Butadiene-Styrene)/Poly (N-Isopropylacrylamide) Hydrogel Elastomer with Interpenetrating Polymeric Networks. Macromol. Mater. Eng..

[70] Yang X., Zhou T., Ren B., Hursthouse A., Zhang Y. (2018). Removal of Mn (II) by sodium alginate/graphene oxide composite double-network hydrogel beads from aqueous solutions. Sci. Rep..

[71] Raia N.R., Jia D., Ghezzi C.E., Muthukumar M., Kaplan D.L. (2020). Characterization of silk-hyaluronic acid composite hydrogels towards vitreous humor substitutes. Biomaterials.

[72] Hasturk O., Jordan K.E., Choi J., Kaplan D.L. (2020). Enzymatically crosslinked silk and silk-gelatin hydrogels with tunable gelation kinetics, mechanical properties and bioactivity for cell culture and encapsulation. Biomaterials.

[73] Gao Y., Kong W., Li B., Ni Y., Yuan T., Guo L., Lin H., Fan H., Fan Y., Zhang X. (2018). Fabrication and characterization of collagen-based injectable and self-crosslinkable hydrogels for cell encapsulation. Colloids Surf. B Biointerfaces.

[74] Matera D.L., DiLillo K.M., Smith M.R., Davidson C.D., Parikh R., Said M., Wilke C.A., Lombaert I.M., Arnold K.B., Moore B.B. (2020). Microengineered 3D pulmonary interstitial mimetics highlight a critical role for matrix degradation in myofibroblast differentiation. Sci. Adv..

[75] Utech S., Boccaccini A.R. (2016). A review of hydrogel-based composites for biomedical applications: Enhancement of hydrogel properties by addition of rigid inorganic fillers. J. Mater. Sci..

[76] Horkay F., Basser P.J. (2020). Composite Hydrogel Model of Cartilage Predicts Its Load-Bearing Ability. Sci. Rep..

[77] Chen X., Song Z., Li S., Thang N.T., Gao X., Gong X., Guo M. (2020). Facile one-pot synthesis of self-assembled nitrogen-doped carbon dots/cellulose nanofibril hydrogel with enhanced fluorescence and mechanical properties. Green Chem..

[78] Gong J., Wang L., Wu J., Yuan Y., Mu R.-J., Du Y., Wu C., Pang J. (2019). The rheological and physicochemical properties of a novel thermosensitive hydrogel based on konjac glucomannan/gum tragacanth. LWT.

[79] Burak J., Grela K.P., Pluta J., Karolewicz B., Marciniak D.M. (2018). Impact of sterilisation conditions on the rheological properties of thermoresponsive pluronic f-127-based gels for the ophthalmic use. Acta Pol. Pharm.-Drug Res..

[80] Mendoza L., Batchelor W., Tabor R.F., Garnier G. (2018). Gelation mechanism of cellulose nanofibre gels: A colloids and interfacial perspective. J. Colloid Interface Sci..

[81] Nadgorny M., Xiao Z., Connal L.A. (2017). 2D and 3D-printing of self-healing gels: Design and extrusion of self-rolling objects. Mol. Syst. Des. Eng..

[82] Deng S., Li X., Yang W., He K., Ye X. (2018). Injectable in situ cross-linking hyaluronic acid/carboxymethyl cellulose based hydrogels for drug release. J. Biomater. Sci. Polym. Ed..

[83] Balakrishnan B., Soman D., Payanam U., Laurent A., Labarre D., Jayakrishnan A. (2017). A novel injectable tissue adhesive based on oxidized dextran and chitosan. Acta Biomater..

[84] Bertasa M., Dodero A., Alloisio M., Vicini S., Riedo C., Sansonetti A., Scalarone D., Castellano M. (2020). Agar gel strength: A correlation study between chemical composition and rheological properties. Eur. Polym. J..

[85] Bian H., Jiao L., Wang R., Wang X., Zhu W., Dai H. (2018). Lignin nanoparticles as nano-spacers for tuning the viscoelasticity of cellulose nanofibril reinforced polyvinyl alcohol-borax hydrogel. Eur. Polym. J..

[86] Antoniuk I., Kaczmarek D., Kardos A., Varga I., Amiel C. (2018). Supramolecular Hydrogel Based on pNIPAm Microgels Connected via Host–Guest Interactions. Polymers.

[87] Garcia-Hernandez A., Lobato-Calleros C., Vernon-Carter E., Sosa-Hernandez E., Alvarez-Ramirez J. (2017). Effects of clay concentration on the morphology and rheological properties of xanthan gum-based hydrogels reinforced with montmorillonite particles. J. Appl. Polym. Sci..

[88] Fariña M., Torres M.D., Moreira R. (2019). Starch hydrogels from discarded chestnuts produced under different temperature-time gelatinisation conditions. Int. J. Food Sci. Technol..

[89] Ajovalasit A., Sabatino M.A., Todaro S., Alessi S., Giacomazza D., Picone P., Di Carlo M., Dispenza C. (2018). Xyloglucan-based hydrogel films for wound dressing: Structure-property relationships. Carbohydr. Polym..

[90] Gu S., Cheng G., Yang T., Ren X., Gao G. (2017). Mechanical and Rheological Behavior of Hybrid Cross-Linked Polyacrylamide/Cationic Micelle Hydrogels. Macromol. Mater. Eng..

[91] Basu A., Lindh J., Ålander E., Strømme M., Ferraz N. (2017). On the use of ion-crosslinked nanocellulose hydrogels for wound healing solutions: Physicochemical properties and application-oriented biocompatibility studies. Carbohydr. Polym..

[92] Jamburidze A., De Corato M., Huerre A., Pommella A., Garbin V. (2017). High-frequency linear rheology of hydrogels probed by ultrasound-driven microbubble dynamics. Soft Matter.

[93] Farrell K., Joshi J., Kothapalli C.R. (2017). Injectable uncrosslinked biomimetic hydrogels as candidate scaffolds for neural stem cell delivery. J. Biomed. Mater. Res. Part A.

[94] Hashemnejad S.M., Kundu S. (2017). Probing gelation and rheological behavior of a self-assembled molecular gel. Langmuir.

[95] Sood N., Bhardwaj A., Mehta S., Mehta A. (2016). Stimuli-responsive hydrogels in drug delivery and tissue engineering. Drug Deliv..

[96] Narayanaswamy R., Torchilin V.P. (2019). Hydrogels and their applications in targeted drug delivery. Molecules.

[97] Caló E., Khutoryanskiy V. (2015). Biomedical applications of hydrogels: A review of patents and commercial products. Eur. Polym. J..

[98] Chen M.H., Wang L.L., Chung J.J., Kim Y.-H., Atluri P., Burdick J.A. (2017). Methods to assess shear-thinning hydrogels for application as injectable biomaterials. ACS Biomater. Sci. Eng..

[99] Afzal S., Maswal M., Dar A.A. (2018). Rheological behavior of pH responsive composite hydrogels of chitosan and alginate: Characterization and its use in encapsulation of citral. Colloids Surf. B Biointerfaces.

[100] Dreifke M.B., Jayasuriya A.A., Jayasuriya A.C. (2015). Current wound healing procedures and potential care. Mater. Sci. Eng. C.

[101] Kamoun E.A., Kenawy E.-R.S., Chen X. (2017). A review on polymeric hydrogel membranes for wound dressing applications: PVA-based hydrogel dressings. J. Adv. Res..

[102] Azimi B., Maleki H., Zavagna L., De La Ossa J.G., Linari S., Lazzeri A., Danti S. (2020). Bio-Based Electrospun Fibers for Wound Healing. J. Funct. Biomater..

[103] Morariu S., Bercea M., Brunchi C.-E. (2018). Influence of Laponite RD on the properties of poly(vinyl alcohol) hydrogels. J. Appl. Polym. Sci..

[104] Yin F., Lin L., Zhan S. (2019). Preparation and properties of cellulose nanocrystals, gelatin, hyaluronic acid composite hydrogel as wound dressing. J. Biomater. Sci. Polym. Ed..

[105] Quah S.P., Smith A.J., Preston A.N., Laughlin S.T., Bhatia S.R. (2018). Large-area alginate/PEO-PPO-PEO hydrogels with thermoreversible rheology at physiological temperatures. Polymer.

[106] Drury J.L., Mooney D.J. (2003). Hydrogels for tissue engineering: Scaffold design variables and applications. Biomaterials.

[107] Sun Y., Nan D., Jin H., Qu X. (2020). Recent advances of injectable hydrogels for drug delivery and tissue engineering applications. Polym. Test..

[108] Alarake N.Z., Frohberg P., Groth T., Pietzsch M. (2017). Mechanical properties and biocompatibility of in situ enzymatically cross-linked gelatin hydrogels. Int. J. Artif. Organs.

[109] Alinejad Y., Adoungotchodo A., Grant M.P., Epure L.M., Antoniou J., Mwale F., Lerouge S. (2019). Injectable chitosan hydrogels with enhanced mechanical properties for nucleus pulposus regeneration. Tissue Eng. Part A.

[110] Dorishetty P., Balu R., Athukoralalage S.S., Greaves T.L., Mata J.P., de Campo L., Saha N., Zannettino A.C.W., Dutta N.K., Choudhury N.R. (2020). Tunable Biomimetic Hydrogels from Silk Fibroin and Nanocellulose. ACS Sustain. Chem. Eng..

[111] Katoch A., Choudhury A.R. (2020). Understanding the rheology of novel guar-gellan gum composite hydrogels. Mater. Lett..

[112] Rizzo C., Andrews J.L., Steed J.W., D’Anna F. (2019). Carbohydrate-supramolecular gels: Adsorbents for chromium(VI) removal from wastewater. J. Colloid Interface Sci..

[113] Regubalan B., Pandit P., Maiti S., Nadathur G.T., Mallick A. (2018). Potential bio-based edible films, foams, and hydrogels for food packaging. Bio-Based Materials for Food Packaging.

[114] Murali S., Kumar S., Koh J., Seena S., Singh P., Ramalho A., Sobral A.J. (2019). Bio-based chitosan/gelatin/Ag@ ZnO bionanocomposites: Synthesis and mechanical and antibacterial properties. Cellulose.

[115] Sun S., Xiao Q.-R., Zhou X., Wei Y.-Y., Shi L., Jiang Y. (2018). A bio-based environment-friendly membrane with facile preparation process for oil-water separation. Colloids Surf. A Physicochem. Eng. Asp..

[116] Wang G., Zhang Q., Wang Q., Zhou L., Gao G. (2021). Bio-Based Hydrogel Transducer for Measuring Human Motion with Stable Adhesion and Ultrahigh Toughness. ACS Appl. Mater. Interfaces.

